# The Relieving Effects of BrainPower Advanced, a Dietary Supplement, in Older Adults with Subjective Memory Complaints: A Randomized, Double-Blind, Placebo-Controlled Trial

**DOI:** 10.1155/2016/7898093

**Published:** 2016-04-11

**Authors:** Jingfen Zhu, Rong Shi, Su Chen, Lihua Dai, Tian Shen, Yi Feng, Pingping Gu, Mina Shariff, Tuong Nguyen, Yeats Ye, Jianyu Rao, Guoqiang Xing

**Affiliations:** ^1^Department of Community Health and Family Medicine, School of Public Health, Shanghai Jiao Tong University, Shanghai 200025, China; ^2^School of Public Health, Shanghai University of TCM, Shanghai 201203, China; ^3^Si-Tang Community Health Service Center of Shanghai, Shanghai 200431, China; ^4^Department of Emergency Medicine, Xin Hua Hospital Affiliated to Shanghai Jiao Tong University School of Medicine, Shanghai 200092, China; ^5^Southern California Kaiser Sunset, 4867 Sunset Boulevard, Los Angeles, CA 90027, USA; ^6^Department of Research, DRM Resources, 1683 Sunflower Avenue, Costa Mesa, CA 92626, USA; ^7^Maryland Population Research Center, University of Maryland, College Park, MD 20742, USA; ^8^Department of Pathology and Laboratory Medicine, David Geffen School of Medicine, University of California at Los Angeles, Los Angeles, CA 90095, USA; ^9^Imaging Institute of Rehabilitation and Development of Brain Function, North Sichuan Medical University, Nanchong Central Hospital, Nanchong 637000, China; ^10^Lotus Biotech.com LLC, John Hopkins University-MCC, 9601 Medical Center Drive, Rockville, MD 20850, USA

## Abstract

Subjective memory complaints (SMCs) are common in older adults that can often predict further cognitive impairment. No proven effective agents are available for SMCs. The effect of BrainPower Advanced, a dietary supplement consisting of herbal extracts, nutrients, and vitamins, was evaluated in 98 volunteers with SMCs, averaging 67 years of age (47–88), in a randomized, double-blind, placebo-controlled trial. Subjective hypomnesis/memory loss (SML) and attention/concentration deficits (SAD) were evaluated before and after 12-week supplementation of BrainPower Advanced capsules (*n* = 47) or placebo (*n* = 51), using a 5-point memory questionnaire (1 = no/slight, 5 = severe). Objective memory function was evaluated using 3 subtests of visual/audio memory, abstraction, and memory recall that gave a combined total score. The BrainPower Advanced group had more cases of severe SML (severity *⩾* 3) (44/47) and severe SAD (43/47) than the placebo group (39/51 and 37/51, < 0.05, < 0.05, resp.) before the treatment. BrainPower Advanced intervention, however, improved a greater proportion of the severe SML (29.5%)(13/44) (*P* < 0.01) and SAD (34.9%)(15/43)(*P* < 0.01) than placebo (5.1% (2/39) and 13.5% (5/37), resp.). Thus, 3-month BrainPower Advanced supplementation appears to be beneficial to older adults with SMCs.

## 1. Introduction

Memory is the ability of an individual to record, retain, and recall sensory stimuli, events, and information over short and long periods of time. Deficits in memory function can compromise one's quality of life and ability to work.

Hypomnesis/forgetfulness/memory decline can occur with aging or as results of subhealth conditions [1]. Complaints about memory impairment, or subjective memory complaints (SMCs), are common in elderly people but are rarely detected by clinicians using objective memory function tests due to the subtle and heterogeneous nature of SMC [2–5]. SMCs are a criterion of mild cognitive impairment (MCI), which is common in older adults and in people who have experienced subhealth conditions [6–12].

MCI is an intermediary status between normal aging and prodromal memory decline [6, 13]. Individuals with MCI appear to have intact general cognitive function and activities of daily living, but their memory is impaired for normal age. The prevalence of MCI is estimated at 3%–19% in adults older than 65 years and 15% in adults older than 75 years [14–18]. Less than half of people with MCI are stable or able to reverse back to normal memory function again within 5 years [19–21].

SMC and MCI are often associated with a decline in episodic memory (a recollection of specific past events) [22–24], which is most frequently found in those with the amnesic subtype MCI [22–28]. Positron emission tomography (PET) brain image studies show that people with SMC are characterized with elevated brain beta-amyloid [25–27]. Increased amyloid deposition has an early and subclinical impact on cognition that precedes hypometabolism [28] and impairs blood vessel functions [29] that could contribute to increased inflammation in amnestic mild cognitive impairment [30].

Latent insufficiency of cerebrovascular circulation and loss of phospholipid asymmetry may underlie early manifestation of SMC and MCI [31] and could be a risk factor for episodic nonspecific complaints of mild cognitive deficit, regional hypoperfusion, and hypometabolism [32–38].

Episodic memory processes depend on the integrity of the medial temporal lobe, hippocampus, the posterior parietal cortex, and lateral prefrontal cortex (PFC) [33–35]. Imaging studies have shown an asymmetric hemispheric encoding/retrieval (HERA) pattern in young adults where the left PFC and temporo-occipital cortex are involved in encoding and the right PFC is involved in retrieval of the stored information [36–39]. During normal aging, PFC activation becomes less asymmetric during memory tasks. Normal or high-performing older adults balance age-related neural decline through neuroplasticity, which reorganizes neurocognitive networks. The subnormal or low-performing older adults use a network similar to young adults, but inefficiently [36, 40].

Abnormal cholinergic and glutamatergic neurotransmissions are thought to be involved in SMC and MCI [41–43]. Cognitive decline in older adults is associated with a loss of cholinergic function (cholinergic hypofunction) including a reduction in choline acetyltransferase (ChAT), muscarinic and nicotinic acetylcholine receptor binding sites, and concentrations of acetylcholine in the synaptic clefts [43, 44]. Glutamatergic overstimulation (excitotoxicity) of the postsynaptic NMDA receptors could also lead to memory impairment [45].

Currently, there are no effective and safe pharmaceutical drugs for SMC and MCI. Prevention of the progression of the symptomatic development could be the best strategy [46]. Although acetylcholinesterase inhibitors (AChEIs) and N-methyl D-aspartate (NMDA) receptor antagonists have been used for treatment of people with varying degree of memory deficits, their effects on cognition and memory improvement are often negative [47–54]. A portion of people with MCI, however, may respond favorably to AChEIs (15%–35%) and NMDA receptor antagonists (30%) [55–57], but usually after high doses or long treatment, and with potential adverse effects, such as nausea, vomiting, diarrhea, headache, hypertension, and hepatotoxicity [58–64].

A variety of plant-derived compounds have been studied as potential enhancers of memory and cognitive function [65] with potential mechanisms on (1) modulation of neuronal membrane integrity; (2) modulation of the cholinergic system through inhibition of acetylcholinesterase (AChE) or stimulation of muscarinic and nicotinic receptors; (3) neuroprotection against NMDA receptor excitotoxicity; (4) anti-inflammatory and antioxidant activities; (5) improved cerebral blood flow and microperfusion.

Table 1 lists some herbal extracts and compounds that are active ingredients of BrainPower Advanced, the formulation used in this pilot study. BrainPower Advanced is a dietary supplement formulated to support healthy memory and cognitive function in adults. Its key ingredients include extracts of* Ginkgo biloba* (flavonoids, terpenoids, and terpene lactones),* Camellia sinensis* leaf (tea polyphenols),* Catharanthus roseus *(vinpocetine), kola nut (*Cola nitida*, caffeine),* Huperzia serrata* (hup A and hup B), phosphatidylserine (PS), L-tyrosine, L-pyroglutamic acid, acetyl-L-carnitine, choline bitartrate, L-glutamine, L-phenylalanine, L-cysteine, vitamin B6, and vitamin B12 (Table 1).

PS is a key integrative phospholipid component of neuronal cell membranes and represents 15% of the total phospholipid pool. PS acts to maintain membrane integrity and neuroplasticity, buffer oxidative stress, facilitate neurotransmitter release, and increase brain glucose metabolism [66–69]. Phospholipid deficits in neuronal membranes are involved in age-related brain-structural and cognitive decline. A decline of PS and other phospholipids in neuronal membranes has been associated with memory impairment and cognitive deficits whereas dietary PS and phospholipid supplements have prevented or reversed such deficits [70–75]. PS supplementation improved learning capacity and memory in rodents [76, 77] and improved physical and mental performance such as long-term memory and recognition in elderly people with cognitive decline [73, 78–80], in stressed young adults [81], and in children with attention deficit hyperactivity disorder [82].

Vinpocetine (ethyl apovincaminate) is synthesized from the alkaloid vincamine, an extract from the leaf of the lesser periwinkle plant (*Catharanthus roseus*). Vinpocetine has been used widely in Japan and Europe for the treatment of cognitive decline since the late 1970s. Vinpocetine can improve cerebral blood flow and glucose metabolism in the thalamus and basal ganglia and the occipital, parietal, and temporal cortex [83–86]. Previous studies have confirmed that vinpocetine can inhibit beta-amyloid-induced activation of NF-*κ*B, inflammation, and cytokine production and interferes with many stages of the ischemic cascade: ATP depletion, activation of voltage-sensitive Na(+) and Ca(++) channels, and glutamate and free-radical release (antioxidant activity) [87–91]. Vinpocetine treatment for 18 months significantly improved cognitive functions, overall health status, and quality of life in people with chronic cerebral hypoperfusion [92]. Vinpocetine is considered prophylactic for MCI [92–94].

Huperzine A (hup A), is a quinolizidine-related alkaloid isolated from* Huperzia serrata* (Thunb.) Trevis. Hup A is a competitive, reversible, and well-tolerated inhibitor of AChE that is more potent than current memory-promoting agents (donepezil, rivastigmine, and galantamine) [95–98]. Hup A is also a competitive NMDA receptor inhibitor and an antagonist of A*β*-induced neurotoxicity [99–108]. It has significant antioxidative, antiapoptotic, and neuroprotective effects [100]. Hup A has been used for enhancing memory and mental function in healthy people [101] and in animal models of cognitive deficits [97] without showing any obvious serious adverse effects [102]. Other studies suggest, however, that hup A has limited effects on cognition. One recent multicenter, randomized, placebo-controlled trial showed that a dose of 0.4 mg daily, but not 0.2 mg daily, of hup A was effective in improving cognition in people with moderate cognition decline [103].

The active components of the leaf extract of* Ginkgo biloba* (EGb) consist of flavonoids, terpenoids, and terpene lactones (ginkgolides and bilobalide). EGb has long been used in traditional Chinese medicine [104, 105]. EGb has also been prescribed for memory and concentration complaints in Germany and France since the 1960s [106, 107]. EGb protects neuronal cell membranes and mitochondria from free-radical damage [108]; reduces the aggregation and toxicity of beta-amyloid [109, 110]; promotes hippocampal neurogenesis [111–116]; decreases blood viscosity [117, 118]; enhances microperfusion [119]; improves neurotransmission of glutamatergic [120–122], dopaminergic, and cholinergic systems [123, 124]; and improves learning and memory in animal models [125–129].

Recent meta-analyses of multiple randomized, controlled trials suggest that EGb may be effective for reversing or delaying age-related memory deficits [130–135]. However, two recent large-scale studies, conducted in more than 6000 participants aged 70 years and over [136, 137], failed to confirm such effects. One explanation is that, due to the particularly long incubation phase of severe memory decline (20–30 years), a quick reduction in the incidence of severe memory deficits by EGb is unrealistic, or EGb is only effective in subjects at early stages of memory decline such as SMC and/or MCI, but not in advanced stage of memory deficits. To support this, short-term and acute EGb administration could be used to enhance specific memory and cognitive functions in cognitively intact older, middle-aged, and younger healthy volunteers [138]. In addition, coadministration of EGb and PS improved secondary memory and the speed of memory task performance [125, 126].

Other studies show that deficits or dysregulation in brain metabolism is linked with aging-related cognition decline and that decline could be reversed through nutrients and vitamin supplements such as acetyl-L-carnitine, vitamin B6, and vitamin B12 [139–151]. L-Tyrosine is a precursor to L-dopa and catecholamines, an imbalance of which may be involved in cognitive dysfunction [127, 128].

The preventive effects of coffee, tea, and caffeine consumption on late-life cognitive decline and dementia have been extensively reviewed recently [129]. Besides the well-known short-term enhancing effects, some studies examined the long-term effects and showed that coffee, tea, and caffeine consumption could protect against late-life cognitive impairment/decline and dementia. These findings, however, are still considered preliminary [129].

Green tea (*Camellia sinensis*) extract enhances parietofrontal connectivity during working memory processing [152] and protects against okadaic acid-induced acute learning and memory impairments in rats [153]. A recent review suggests that the green tea constituent theanine could have therapeutic effects against psychiatric and neurodegenerative disorders including mild cognitive impairment and dementia through multiple mechanisms such as inhibition of NMDA-induced neurotoxicity, enhanced brain GABA and glycine content, and enhanced BDNF-related neuroprotection [154]. Most of these mechanistic studies, however, were done on animals.

Coadministration of multiple medicinal herbal ingredients is a common practice in traditional Chinese medicine. The main goal of such practices is to potentiate the bioavailability, activity, and efficacy of the key therapeutic ingredients and to minimize or antagonize potential toxicities associated with the ingredients.

The objective of this exploratory, randomized, placebo-controlled trial was to evaluate the safety and effectiveness of short-term administration of BrainPower Advanced, a multi-ingredient dietary supplement, on SMCs in older adults.

## 2. Methods

### 2.1. Samples and Participants Recruitment

This study was conducted between December 15, 2011, and April 10, 2012, at the Si Tang Community Health Service Center in Baoshan district in Shanghai, China. Community volunteers of older adults were recruited by self-referral in response to media coverage and word of mouth. All study procedures were conducted in accordance with the Helsinki Declaration of 1975 and were approved by the Shanghai Jiao Tong University Medical Center Institutional Review Board. Informed consent was obtained from all participants prior to enrollment into the study.

Subjects who met the following criteria were eligible for the study:* inclusion criteria*: (1) healthy males or females at least 50 years of age; (2) having self-reported hypomnesis, forgetfulness, memory loss, or impaired attention/concentration as determined by a standard medical examination questionnaire;* exclusion criteria*: (1) having been diagnosed with neurological diseases such as Alzheimer's Disease, Parkinson's Disease, migraine, and epilepsy; (2) having taken any medication likely to affect brain and nervous system function such as L-dopa, MAO inhibitors, modafinil, and amphetamine, within 30 days before the start of the trial; (3) having a history of severe cerebral-cardiovascular events (myocardial infarction, cerebrovascular disorder, acute coronary syndrome, or other cerebrovascular diseases); (4) other severe medical conditions (severe diseases of the liver, kidneys, or lungs; malignant tumors; motor impairment; dysphonia; visual impairment; etc.).

### 2.2. Randomization and Blindness

Participants were randomly assigned to the BrainPower Advanced treatment group or placebo treatment group. The randomization was performed using a predetermined randomization code which was generated by a random number generator. The numbers generated were placed in sealed envelopes and a serial number was assigned to each envelope according to the sequence of allocation of the randomized number. Each envelope was then opened sequentially according to the admission sequence of subjects.

Trial participants and community doctors were both blinded from the treatment (double-blind trial). Of the 101 enrolled participants, 98 participants completed the 12-week follow-up, including 47 subjects in the BrainPower Advanced group and 51 subjects in the placebo group. Three subjects withdrew from the study (2 due to symptoms of diarrhea and 1 due to family objection).

The participants received similar-looking capsules in color-coded bottles (white bottles for BrainPower Advanced and yellow bottles for placebo). Neither the subjects nor the medical doctors, including the study principal investigator (Rong Shi), knew the specific color code until the end of the study. Both the BrainPower Advanced capsules and the placebo (which was mainly composed of flour) were manufactured and supplied by Robinson Pharma, Inc. (Costa Mesa, California, USA).

Each participant was instructed to take 2 capsules with meals daily for 12 weeks and a new batch of supplements was dispensed every 4 weeks during follow-up sessions. Changes in subjective hypomnesis/memory loss and attention/concentration deficits were recorded using a self-administrated medical questionnaire. Memory function capacity was evaluated using a subset of tester-administered memory tests before and after the 12-week intervention. All participants were followed up each month in order to check compliance and adverse effects.

### 2.3. Evaluation of Subjective Memory Complaints

Two aspects of subjective memory complaints, that is, subjective hypomnesis/forgetfulness/memory loss (SML) and subjective attention/concentration deficits (SAD), were screened using a self-administered 5-point scale (1 = no symptoms or occasional slight symptoms complaints; 2 = slight/mild symptom complaints; 3 = moderate severe symptom complaints; 4 = severe symptom complaints; 5 = very severe symptom complaints) included in a medical questionnaire that included the demographics and medication history of the participants. Because relatively few participants scored 1 and 2 points in the memory questionnaire, participants who scored 1 and 2 points were combined as the “slight symptom” group, and participants who scored 3, 4, and 5 points were combined as the “severe symptom” group for further statistical analysis.

### 2.4. Evaluation of Objective Memory Function

The short-term working memory of the participants was evaluated using three simple tester-administered subtests constructed and validated for the Chinese population [155].

*(1) Visual/Auditory Memory (1 Point for Each Correct Answer, a Maximal Total of 5 Points)*. Subjects were allowed to watch 5 film clips in 5 minutes (1 minute per film clip). The films were selected from a pool of popular old Chinese films that all participants should have been familiar with. The subjects were then asked to recall the correct order and content of the films based on the movie listings provided by the tester.

*(2) Abstracting (1 Point for Each Correct Answer, a Maximal Total of 5 Points)*. Subjects were shown 5 different pictures/images of cartoon characters (such as a policeman, soldier, medical doctor, nurse, taxi driver, bus driver, school teacher, university professor, cook, waitress, and tour guide) on a computer screen and were asked to recall them immediately.

*(3) Memory Recall (1 Point for Each Correct Answer, a Maximal Total of 5 Points)*. Subjects were shown 5 consecutive pictures of famous historical landscapes located in different Chinese cities and were asked to recall the pictures' contents and their locations.

### 2.5. Statistics Analysis

EpiData 3.02 software was used for the establishment of the database. SPSS 20.0 software was used for statistical analysis. Group data were presented as the mean ± s.d. Mean differences of the variables between the BrainPower Advanced and placebo groups were compared using Student's *t*-test for variables with normal distribution or using nonparametric tests for variables with nonnormal distributions. Ridit scoring test, which is a nonparameter test for comparing two or more sets of ordered qualitative data, was used for evaluating the changes in the symptom severity score of SML and SAD in response to the intervention. The alpha level of *P* > 0.05 was chosen as being statistically significant. All *P* values reported were 2-sided.

## 3. Results

### 3.1. Participants' Characteristics

The baseline characteristics of age and gender and histories of alcohol intake, disease, and medication of the participants are shown in Table 2. There were 14 males (29.8%) and 33 females (70.2%) in the BrainPower Advanced group and 17 males (33.3%) and 34 females (66.7%) in the placebo group. The gender distribution between the two groups was not significantly different (*χ* ^2^ = 0.142, *P* > 0.05), with females accounting for more than two-thirds of the total participants. The overall average age of all participants was 67.1 ± 10.5, with no significant difference found between the BrainPower Advanced group (69.1 ± 9.5 years) and the placebo group (65.2 ± 11.1 years) (*t* = 1.839, *P* > 0.05). The BrainPower Advanced and placebo groups also showed similar patterns of alcohol intake history (*χ* ^2^ = 0.542, *P* > 0.05). However, a greater proportion of the participants in the BrainPower Advanced group had disease history (39/47, 83%) and medication history (35/47, 74.5%) compared to the placebo group (31/51, 60.8%, *χ* ^2^ = 5.904, *P* < 0.05 and 23/51, 45.1%, *χ* ^2^ = 8.734, *P* < 0.01, resp.).

### 3.2. Subjective Memory Complaints (SMC)

The mean value, distribution pattern, and the differences between the BrainPower Advanced group and placebo group in the severity level of subjective hypomnesis/forgetfulness/memory loss (SML) and subjective attention/concentration deficit (SAD) before and after the 12-week intervention are shown in Tables 3, 4(a), and 5(a).

The baseline symptom severities of SML and SAD in the BrainPower Advanced group (3.77 ± 0.89; 3.62 ± 0.99, resp.) were about 10% greater than those of the placebo group (3.43 ± 1.19; 3.25 ± 1.35, resp.) (Table 3). These differences in SML and SAD between the two groups disappeared after the 12-week intervention, primarily due to a greater proportion of the BrainPower Advanced group showing greater reduction of symptom severity than the placebo group (Tables 4(b) and 5(b)). The placebo group had more participants that showed worsened symptom severity than the BrainPower Advanced group.

Ridit scoring test shows a significant and differential reduction in the SML (mean ± s.d. = 0.418 ± 0.236 versus 0.575 ± 0.299, *P* < 0.01) and SAD symptom severity (means = 0.424 ± 0.229 versus 0.612 ± 0.283, *P* < 0.01) after BrainPower Advanced and placebo treatment.  Table 4(b) shows that a total of 34 people reported reduced SML symptom severity after BrainPower Advanced intervention (2 people by 3 points, 12 people by 2 points, and 20 people by 1 point), 12 people reported no change, and 1 person reported worsened symptom severity (by 1 point). In comparison, only 23 people reported reduced SML severity after placebo intervention (1 person by 3 points, 10 people by 2 points, and 12 people by 1 point), 15 people reported no change in symptom severity, and 13 people reported worsened symptom severity (10 people by 1 point, 2 people by 2 points, and 1 person by 3 points).

Similarly, Table 5(b) shows that a total of 36 people reported different reductions in SAD symptom severity after BrainPower Advanced intervention (2 people by 3 points, 14 people by 2 points, and 20 people by 1 point), 9 people reported no change, and 2 people reported worsened symptom severity (by 1 point) (Table 5(b)). After placebo intervention, 20 people reported different reductions in SAD symptom severity (2 people by 3 points, 8 people by 2 points, and 10 people by 1 point), 15 people reported no change, and 16 people reported worsened symptoms (11 people by 1 point, 3 people by 2 points, and 2 people by 3 points).

Because the symptom severity scores are nominal variables and because only few participants of the BrainPower Advanced group scored 1 and 2 points (Tables 4(a) and 5(a)) of SML and SAD, participants with the severity scores of 1 and 2 were combined as the “slight symptom” group and participants with the severity scores of 3, 4, and 5 were combined as the “severe symptom” group for further chi-square test (Tables 4(c) and 5(c)).

It was noted that a greater proportion of the BrainPower Advanced group (44/47, 93.6%) than the placebo group (39/51, 76.5%) had severe SML symptoms (severity scores *⩾* 3), or a smaller proportion of the BrainPower Advanced group had slight SML symptoms (severity scores ⩽ 2) (3/47, 6.4%) than the placebo group (12/51, 23.5%) (Pearson *χ* ^2^ = 5.547, *P* = 0.019) before the start of the intervention (Tables 4(a) and 4(c)). Similar pretreatment differences in severe SAD distribution existed between the BrainPower Advanced (43/47, 91.5%) and placebo groups (37/51, 72.5%) (severity scores *⩾* 3) (Pearson *χ* ^2^ = 5.852, *P* = 0.016) (Tables 5(a) and 5(c)).

After the intervention, however, the BrainPower Advanced group had fewer cases of severe SML (31) and SAD (28) than the placebo group (37 for SML and 32 for SAD, resp.) and the difference in SML and SAD between the BrainPower Advanced and placebo groups was no longer significant (Pearson *χ* ^2^ = 0.5, *P* = 0.479; *χ* ^2^ = 0.104, *P* = 0.748, resp.) (Tables 4(c) and 5(c)).

Placebo group analysis of the combined data showed that the proportion of people with severe SML showed little change after placebo intervention (reduced by 2 people, from 39 to 37, *P* > 0.05) whereas the proportion of people with severe SML dropped significantly after BrainPower Advanced intervention (reduced by 13 people, from 44 to 31, *P* < 0.001) (Table 4(c)). Similarly, the proportion of severe SAD did not change significantly after placebo intervention (reduced by 5 people, from 37 to 32, *P* > 0.05) whereas the proportion of severe SAD decreased significantly after BrainPower Advanced intervention (reduced by 15 people, from 43 to 28, *P* < 0.001) (Table 5(c)).

### 3.3. Memory Function Test Scores

The memory function test focused on 3 subareas: visual/auditory impression memory, abstract thinking, and immediate memory recall. The subtest scores and the combined total test scores are shown in Tables 6(a) and 6(b), respectively. The BrainPower Advanced group had about 10% lower baseline scores than placebo group in audio/visual memory (2.28 ± 1.06 versus 2.51 ± 0.81), abstract thinking (2.40 ± 1.08 versus 2.73 ± 1.08), and combined total memory function scores (6.85 ± 2.46 versus 7.37 ± 2.32), but similar scores in memory retrieval (2.17 ± 1.15 versus 2.18 ± 1.14). These baseline differences disappeared after the 12-week intervention (3.43 ± 0.83 versus 3.41 ± 1.00, 3.45 ± 0.75 versus 3.47 ± 0.92, 9.66 ± 1.85 versus 9.39 ± 1.98, and 2.81 ± 0.92 versus 2.47 ± 0.83, resp.). Both the BrainPower Advanced and the placebo groups produced significant improvements (129%–150%) (*P* < 0.01, each) of the subtests and combined total scores in the older adults, with the BrainPower Advanced group producing better improvements (1.50-, 1.35-, 1.43-, and 1.29-fold relative to the baseline values of audio/visual memory, abstract thinking, memory retrieval, and combined total function test scores) than the placebo group (1.35-, 1.27-, 1.13-, and 1.27-fold, resp.) (Tables 6(a) and 6(b)).

Ridit scoring test showed that the improvement in the combined total scores of the memory function subtests was significantly better after the BrainPower Advanced intervention (mean = 0.56 ± 0.287) than after the placebo intervention (0.445 ± 0.276) (*P* < 0.05) (Table 6(c)).

## 4. Discussion

In this exploratory randomized, double-blind, placebo-controlled study, we evaluated the effectiveness of a proprietary dietary supplement, BrainPower Advanced, in older adults with SMCs. The results show that BrainPower Advanced intervention for 12 weeks was safe and effective in improving the symptoms of SMC in older adults.

SMCs are defined as self-awareness of memory loss that can be assessed by a simple “yes” or “no” questionnaire but are often not detected by clinicians using objective memory scales [2]. SMC is also a predictor of mild cognitive impairment (MCI) and of future cognitive decline [156, 157]. There are studies showing that people with SMC are 3–6 times more likely to develop MCI than people without SMC [158–162].

Because SMCs are often reversible, improvement of SMC would represent a good opportunity to intervene prodromal MCI and cognitive decline. So far, no proven agents are available for SMC. BrainPower Advanced is a dietary supplement based on polyherbal extracts, nutrients, and vitamins. Previous studies have shown that the active ingredients of BrainPower Advanced could potentially improve cognitive function in different age groups of healthy people in the presence or absence of SMCs through various mechanisms including anti-inflammation, improved cerebral blood flow and perfusion, and improved glucose/energy metabolism (Table 1).

In the present study, BrainPower Advanced treatment reversed 13 of the 44 (29.5%) cases of severe SML and 15 of the 43 (34.9%) cases of severe SAD. In contrast, placebo treatment reversed only 2 of the 39 (5.1%) cases of severe SML (*P* > 0.05) and 5 of the 37 (13.5%) cases of severe SAD (*P* > 0.05). Furthermore, a greater proportion of the participants showed various degrees of symptom improvement after BrainPower Advanced intervention (72.3%, 34/47 for SML and 76.6%, 36/47 for SAD) than after placebo treatment (49%, 25/51 for SML and 39.2%, 20/51 for SAD).

In contrast, a greater proportion of participants in the placebo group reported worsened symptoms of SML (25.5%, 13/51) and SAD (31.4%, 16/51) or no change in SML (29.4%, 15/51) and SAD (29.4%, 15/51) compared to the BrainPower Advanced group that reported worsened SML (2.1%, 1/47) and SAD (2%, 2/47) or no change in SML (25.5%, 12/47) and SAD (19.1%, 9/47) after the intervention.

The 29.5% reversion rate (13/44) of severe SML and 34.9% reversion rate (15/43) of severe SAD by BrainPower Advanced are comparable to the reported 15%–35% response rate of MCI to AChEIs treatment and the 30% response rate of MCI to NMDA receptor antagonist treatment [139]. Given that there is no standard anti-SMC treatment yet, the current results are very promising [140–142].

These results suggest that BrainPower Advanced is not only effective in reducing a significant portion (about 30%) of severe SMC and in reducing symptom severity in about 70% of the subjects with SMCs, but also effective in reducing the progression or worsening of SMCs. It is noted, however, that 25.5% of the people with severe SML showed no response to BrainPower Advanced intervention.

In this study, no significant differences were found in visual/auditory memory, abstract thinking, and memory retrieval and in the combined total memory function testing scores between the BrainPower Advanced and placebo groups before or after the intervention. Both BrainPower Advanced and placebo interventions resulted in significantly improved memory function performance, albeit with the BrainPower Advanced group showing better improvement than the placebo group at a nonsignificant level.

Because both placebo and BrainPower Advanced intervention enhanced the performance scores of the memory function tests, this raised the possibility that factors other than the intervention per se may be responsible for the improvement. One such possibility is that the postintervention memory test score was unintendedly “enhanced” by the preintervention test. Like any learning activity, practice or repetition of the same learning task would improve performance and produce better scores. This may not mean that the memory was “getting better” but simply reflecting the remembered answers from the last test. There are suggestions that the same memory tests should not be given within a short period of time (3–6 months) or that only the tests taken the first time should be taken seriously as a true measure of memory abilities.

It is the current view that SMC is a subjective experience that cannot be reliably measured by objective memory scales due to the subtle and heterogeneous nature of SMC [2–5]. Nevertheless, more accurate diagnosis of SMC and/or MCI could be corroborated by informants and by using different questionnaires and memory scales before and after an intervention such as the use of Mini-Mental State Examination (MMSE), Six-Item Screener, Subjective Memory Rating Scale (SMRS) and Deterioration Cognitive Observee (DECO) [143], Memory Complaint Questionnaire (MAC-Q), and Subjective Cognitive Decline Questionnaire (SCD-Q) [144]. The updated Wechsler Memory Scale (WMS) also has a battery of subtests for evaluating multiple aspects of learning and memory including immediate and delayed memory for visual working memory and auditory memory [145–148].

There are other limitations of this study. The sample size is too small to detect potential performance differences in the memory function tests. Despite the randomization process, more participants in the BrainPower Advanced group had a history of disease and medication use than the placebo group. Due to the multi-ingredient nature of BrainPower Advanced, it was difficult to determine whether any single ingredient (*Ginkgo biloba*,* Camellia sinensis*, vinpocetine, kola nut, hup A, PS, L-tyrosine, L-pyroglutamic acid, acetyl-L-carnitine, choline bitartrate, L-glutamine, L-phenylalanine, L-cysteine, vitamin B6, or vitamin B12) was primarily responsible for BrainPower Advanced overall effects or, more likely, whether it was a synergistic combination of the ingredients working together that produced the observed results. Because SMCs are heterogeneous and potentially affected by a range of genetic and epigenetic factors including lifestyle [149], daily activity/exercise [150, 151], APOE genotype [163], affective status [164–166], education achievements [11, 167], inflammation, and alcohol use, controlling these factors would allow better understanding of BrainPower Advanced intervention in further studies [168].

## 5. Conclusion

Twelve-week BrainPower Advanced intervention was effective and safe in reducing the progression of symptom severity in older adults with severe SML and SAD. As no proven agents are currently available in reversing or delaying the progression of SMC in the increasing aging population, further well-controlled, large-scale studies could validate if long-term dietary supplementation of combined polyherbal ingredients, nutrients, and vitamins could be an alternative prophylactic strategy for older adults with SMCs.

## Figures and Tables

**Table 1:** Information of the key ingredients of BrainPower Advanced.

Ingredients	Main active compounds	Effects and possible mechanisms of actions
Phosphatidylserine	Phosphatidylserine	Improves cognitive performance in elderly adults with memory deficits
		Enhances cognitive performance in school children and adults
		Restores impaired neuronal calcium and glucose uptake and metabolism in aged brain
		Precursor of neuronal membrane phospholipid that is responsible for neuroplasticity, learning, and memory
		Neuroprotection

*Catharanthus roseus*	Vinpocetine	Enhance memory function in young healthy volunteers and in animals
		Protect against ischemia by improving blood perfusion and cerebral blood flow
		Increase glucose and oxygen consumption, cerebral ATP, and cAMP levels
		Improve cerebral microcirculation by inhibiting platelet aggregation
		Reduce red blood cell deformability and cerebral vascular resistance
		Enhance neurotransmitter production, release, and concentration in the brain
		Block voltage-gated sodium channels and potentiate the neuroprotective effect of adenosine in hypoxia

*Huperzia serrata *(whole plant)	Huperzine A Huperzine B	Inhibitor of AChE and NMDAR
		Inhibitor of b-amyloid neurotoxicity
		Strong antioxidative, antiapoptotic, and neuroprotective activities
		Improve cognition in healthy people
		Reverse or attenuate cognitive deficits in older adults

*Ginkgo biloba*	Flavonoids Terpenoids Terpene lactones (ginkgolides and bilobalide)	Large dose may improve cognition, daily living activities, and mood
		Dose-dependent and specific enhancing effects on memory, cognitive performance, and alertness in healthy adults
		Delay cognitive decline in elderly population
		Potentiate the cognitive-enhancing effects of phosphatidylserine
		Memory improving effect in older people with memory deficits
		Increase blood supply, vasodilation, reduce blood viscosity, balance neurotransmitter systems, and reduce free radicals
		Inhibitor of platelet activating factor

Vitamin B6	Pyridoxal, pyridoxamine, and pyridoxine	Homocysteine remethylation cofactor
		Reduce blood homocysteine level which is a risk factor for cerebrovascular disease and neuron toxicity

Vitamin B12		Required for the methylation of homocysteine to methionine and needed for myelin, neurotransmitters, and membrane phospholipids for maintaining the integrity of the central nervous system
		Protects against brain atrophy
		Protects mood and memory function

L-Tyrosine	L-Tyrosine	Promotes protein utilization and enhances IgG antibody induction

L-Pyroglutamic acid	L-Pyroglutamic acid	Makes N-terminal modification in neuronal peptides, hormones and peptides, and analogue/reservoir of glutamate

Green tea extract (*Camellia sinensis*, leaf)	Tea polyphenols (epigallocatechin gallate)	Free-radical scavengers, strong antioxidants, and neuroprotection
		Anti-inflammatory; improve vasodilation and normal blood pressure, normal glucose, and lipid metabolism

Acetyl-L-carnitine	Acetyl-L-carnitine	Regulates neuroplasticity, membrane function, and neurotransmitter release; reduces pain and depression activity at cholinergic neurons; membrane stabilization; and enhancing mitochondrial function

Cola nut extract (kola nitida)	Caffeine Theobromine Theophylline	Decrease brain beta-amyloid

Choline bitartrate		A precursor of acetylcholine, a cholinergic neurotransmitter that declines with advancing age
		Improves auditory and visual word recognition at a dose of 12 g per day for 2 weeks

L-Glutamine		Reduces beta-amyloid and H_2_O_2_-induced stress and DNA damage

L-Phenylalanine		An essential amino acid that can be converted to tyrosine and other excitatory neurotransmitters (dopamine, norepinephrine, and epinephrine)

L-Cysteine		A precursor of the antioxidant glutathione and a flavor

**Table 2:** Demographics and medical history of the participants (*N* = 98).

	Treatment	Male	Female	Subtotal
Gender, *N* (% of subtotal)	BrainPower	14 (29.8%)	33 (70.2%)	47 (100%)
	Placebo	17 (33.3%)	34 (66.7%)	51 (100%)
	Combined	31 (31.6%)	69 (68.4)	98 (100%)

Age, year (mean ± s.d.)	BrainPower	69.68 ± 9.52	68.80 ± 9.60	
	Placebo	64.03 ±10.73	65.81 ± 11.41	
	Combined	69.07 ± 9.48	65.22 ± 11.1	67.07 ± 10.49

Age, year (range)	BrainPower	52.87–82.80	53.18–84.86	
	Placebo	47.28–83.47	49.03–88.43	
	Combined	52.87–84.46	47.28–88.43	47.28–88.43

History of chronic disease, yes/total (%)	BrainPower	39/47 (83.0%)		
	Placebo	31/51 (60.8%)		
	Combined	70/98 (71.4%)		

History of alcohol use, yes/total (%)	BrainPower	5/47 (10.6%)		
	Placebo	8/51 (15.7%)		
	Combined	13/98 (13.3%)		

History of medication, yes/total (%)	BrainPower	35/47 (74.5%)		
	Placebo	23/51 (45.1%)		
	Combined	58/98 (59.2%)		

**Table 3:** Mean values of symptom severity of subjective hypomnesis/memory loss (SML) and subjective attention deficit (SAD) (mean ± s.d.) before and after 12 weeks of BrainPower Advanced and placebo intervention.

Self-reported deficits	Intervention	Before intervention	After intervention	Relative to baseline (= 1)
Subjective hypomnesis/memory loss (SML)	BrainPower	3.77 ± 0.89	2.94 ± 0.94	0.779840849
	Placebo	3.43 ± 1.19	2.88 ± 0.77	0.839650146

Subjective concentration/attention deficit (SAD)	BrainPower	3.62 ± 0.99	2.68 ± 0.89	0.740331492
	Placebo	3.25 ± 1.35	2.92 ± 0.94	0.898461538

**(a):** 

	Distribution of SML symptom severity					
	Group	1, none or slight	2, mild	3, moderate	4, severe	5, very severe
Before intervention	BrainPower group	0 (0%)	3 (6.4%)	16 (34.0%)	17 (36.2%)	11 (23.4%)
	Placebo group	5 (9.8%)	7 (13.7%)	7 (13.7%)	25 (49.0%)	7 (13.7%)
	Total	5 (5.1%)	10 (10.2%)	23 (23.5%)	42 (42.9%)	18 (18.4%)

After intervention	BrainPower group	2 (4.3%)	14 (29.8%)	18 (38.3%)	11 (23.4%)	2 (4.3%)
	Placebo group	2 (3.9%)	12 (23.5%)	27 (52.9%)	10 (19.6%)	0 (0%)
	Total	4 (4.1%)	26 (26.5%)	45 (45.9%)	21 (21.4%)	2 (2.0%)

**(b):** 

Symptom score change	BrainPower group		Placebo group		Total	
	*N*	%	*N*	%	*N*	%
−3	2	4.3	1	2.0	3	3.1
−2	12	25.5	10	19.6	22	22.4
−1	20	42.6	12	23.5	32	32.7
0	12	25.5	15	29.4	27	27.6
+1	1	2.1	10	19.6	11	11.2
+2	0	0.0	2	3.9	2	2.0
+3	0	0.0	1	2.0	1	1.0
Total	47	100.0	51.0	100.0	98	100.0

**(c):** 

Treatment group	Before intervention		After intervention		*P* value of McNemar's test
	No or slight	Severe	No or slight	Severe	
BrainPower group	3 (6.4%)	44 (93.6%)	16 (34.0%)	31 (66.0%)	0.001
Placebo group	12 (23.5%)	39 (76.5%)	14 (27.5%)	37 (72.5%)	0.791
Pearson *χ* ^2^	5.547		0.5		
*P*	0.019		0.479		

**(a):** 

	Distribution of SAD symptom severity					
	Group	1, none or slight	2, mild	3, moderate	4, severe	5, very severe
Before intervention	BrainPower group	1 (2.1%)	3 (6.4%)	16 (34.0%)	19 (40.4%)	8 (17.0%)
	Placebo group	7 (13.7%)	7 (13.7%)	8 (15.7%)	22 (43.1%)	7 (13.7%)
	Total	8 (8.2%)	10 (10.2%)	24 (24.5%)	41 (41.8%)	15 (15.3%)

After intervention	BrainPower group	4 (8.5%)	15 (31.9%)	21 (44.7%)	6 (12.8%)	1 (2.1%)
	Placebo group	2 (3.9%)	17 (33.3%)	16 (31.4%)	15 (29.4%)	1 (2.0%)
	Total	6 (6.1%)	32 (32.7%)	37 (37.8%)	21 (21.4%)	2 (2.0%)

**(b):** 

Symptom score change	BrainPower group		Placebo group		Total	
	*N*	%	*N*	%	*N*	%
−3	2	4.3	2	3.9	4	4.1
−2	14	29.8	8	15.7	22	22.4
−1	20	42.6	10	19.6	30	30.6
0	9	19.1	15	29.4	14	14.3
+1	2	4.3	11	21.6	13	13.3
+2	0	0.0	3	5.9	3	3.1
+3	0	0.0	2	3.9	2	2.0
Total	47	100.0	51	100.0	98	100.0

**(c):** 

Treatment group	Before intervention		After intervention		*P* value of McNemar's test
	No or slight	Severe	No or slight	Severe	
BrainPower group	4 (8.5%)	43 (91.5%)	19 (40.4%)	28 (59.6%)	0.001
Placebo group	14 (27.5%)	37 (72.5%)	19 (37.3%)	32 (62.7%)	0.302
Pearson *χ* ^2^	5.852		0.104		
*P*	0.016		0.748		

**(a):** 

Memory function subtest scores		Before intervention	After intervention	Improvement relative to baseline (= 1)	*t*	*P*
Audio/visual memory	BrainPower	2.28 ± 1.06	3.43 ± 0.83	1.50	−8.247	<0.001
	Placebo	2.51 ± 0.81	3.41 ± 1.00	1.36	−5.014	<0.001
	*t*	−1.232	0.074			
	*P*	0.221	0.941			

Abstracting ability	BrainPower	2.40 ± 1.08	3.45 ± 0.75	1.44	−6.602	<0.001
	Placebo	2.73 ± 1.08	3.47 ± 0.92	1.27	−4.791	<0.001
	*t*	−1.474	0.081			
	*P*	0.144	0.889			

Memory retrieval	BrainPower	2.17 ± 1.15	2.81 ± 0.92	1.29	−3.526	0.001
	Placebo	2.18 ± 1.14	2.47 ± 0.83	1.13	−1.820	0.075
	*t*	−.027	0.410			
	*P*	0.979	0.060			

**(b):** 

	Before intervention	After intervention	*t*	*P*
BrainPower	6.85 ± 2.46	9.66 ± 1.85	8.478	<0.001
Placebo	7.37 ± 2.32	9.39 ± 1.98	6.988	<0.001
*t*	−1.08	0.692		
*P*	0.283	0.491		

**(c):** 

Score change	BrainPower intervention		Placebo control		Total	
	*N*	%	*N*	%	*N*	%
−5	1	2.1	0	0	1	1
−3	0	0	1	2	1	1
−2	1	2.1	2	3.9	3	3.1
−1	1	2.1	5	9.8	6	6.1
0	2	4.3	2	3.9	4	4.1
+1	6	12.8	5	9.8	11	11.2
+2	7	14.9	17	33.3	24	24.5
+3	12	25.5	8	15.7	20	20.4
+4	9	19.1	5	9.8	14	14.3
+5	2	4.3	5	9.8	7	7.1
+6	4	8.5	0	0	4	4.1
+7	2	4.3	1	2	3	3.1
*Total*	*47*	*100.0 *	*51*	*100.0 *	*98*	*100.0 *
